# Total Protein Analysis as a Reliable Loading Control for Quantitative Fluorescent Western Blotting

**DOI:** 10.1371/journal.pone.0072457

**Published:** 2013-08-30

**Authors:** Samantha L. Eaton, Sarah L. Roche, Maica Llavero Hurtado, Karla J. Oldknow, Colin Farquharson, Thomas H. Gillingwater, Thomas M. Wishart

**Affiliations:** 1 Division of Neurobiology, The Roslin Institute and Royal (Dick) School of Veterinary Studies, University of Edinburgh, Edinburgh, United Kingdom; 2 Centre for Integrative Physiology, University of Edinburgh, Edinburgh, United Kingdom; 3 Developmental Biology, The Roslin Institute and Royal (Dick) School of Veterinary Studies, University of Edinburgh, Edinburgh, United Kingdom; 4 Euan MacDonald Centre for Motor Neurone Disease Research, University of Edinburgh, Edinburgh, United Kingdom; Hertie Institute for Clinical Brain Research and German Center for Neurodegenerative Diseases, Germany

## Abstract

Western blotting has been a key technique for determining the relative expression of proteins within complex biological samples since the first publications in 1979. Recent developments in sensitive fluorescent labels, with truly quantifiable linear ranges and greater limits of detection, have allowed biologists to probe tissue specific pathways and processes with higher resolution than ever before. However, the application of quantitative Western blotting (QWB) to a range of healthy tissues and those from degenerative models has highlighted a problem with significant consequences for quantitative protein analysis: how can researchers conduct comparative expression analyses when many of the commonly used reference proteins (e.g. loading controls) are differentially expressed? Here we demonstrate that common controls, including actin and tubulin, are differentially expressed in tissues from a wide range of animal models of neurodegeneration. We highlight the prevalence of such alterations through examination of published “–omics” data, and demonstrate similar responses in sensitive QWB experiments. For example, QWB analysis of spinal cord from a murine model of Spinal Muscular Atrophy using an Odyssey scanner revealed that beta-actin expression was decreased by 19.3±2% compared to healthy littermate controls. Thus, normalising QWB data to β-actin in these circumstances could result in ‘skewing’ of all data by ∼20%. We further demonstrate that differential expression of commonly used loading controls was not restricted to the nervous system, but was also detectable across multiple tissues, including bone, fat and internal organs. Moreover, expression of these “control” proteins was not consistent between different portions of the same tissue, highlighting the importance of careful and consistent tissue sampling for QWB experiments. Finally, having illustrated the problem of selecting appropriate single protein loading controls, we demonstrate that normalisation using total protein analysis on samples run in parallel with stains such as Coomassie blue provides a more robust approach.

## Background

Biochemical analysis using Western blotting is an essential tool in determining relative protein expression in complex biological samples. It is often used in conjunction with mass screening technologies such as proteomics to confirm differential candidate expression in various models of disease. Together with increasingly sophisticated *in vivo* and *in vitro* biological models, quantitative protein expression analyses are frequently being employed in attempts to elucidate the molecular mechanisms regulating cellular form and function in health and disease.

Traditionally, Western blotting with ECL (enhanced chemilluminescence) has been referred to as a semi-quantitative technique due to the lack of cumulative luminescence linearity and limited quantitative reproducibility [1]. With the development of more sensitive fluorescent labelling, which demonstrates a greater quantifiable linear range, sensitivity and stability in comparison to conventional ECL detection [2], analysis of protein expression can be justifiably termed “quantitative”. It is therefore imperative to ensure uniformity of sample loading with an even greater degree of precision to avoid erroneous data acquisition when using these higher resolution tools [3]. The leading company in this market is LICOR and its Odyssey fluorescence imaging scanner appears to have the most significant market penetration at present with 206 instruments currently installed in the UK alone (Personal communication; LICOR technical consultants).

In order to accurately measure protein levels in a sample, “loading control” (LC) proteins are commonly used as internal standards. The loading controls are generally derived from ubiquitously expressed “housekeeping” genes and have been widely used due to their presumed consistent level of expression across a diverse range of samples. Actin and tubulin are two of most frequently used loading controls in biomedical research, however an increasing number of studies have suggested they may be differentially expressed in animal and experimental models [4]–[8]. Furthermore, LCs may also differ in expression from tissue to tissue or following exposure to infectious agents [9]. Therefore, normalising data according to loading control protein expression could further skew results leading to erroneous conclusions.

This study set out to specifically investigate the reliability of loading controls as internal standards, and characterise a robust, reproducible and simple method for normalising protein load when practicing modern quantitative Western blotting. We present data focusing on the expression levels of commonly used loading control proteins in the nervous system, as this is our specific area of research interest. However, we go on to demonstrate the importance of accurate loading controls for research on a broad range of biological tissues and demonstrate that normalisation using total protein analysis (TPA) with stains such as Coomassie and Instant blue provides a more robust baseline for performing QWB experiments.

## Results and Discussion

### Expression Levels of Commonly used “Loading Control” Proteins

In order to obtain an initial estimate for the variability of expression levels of commonly used loading control proteins in experimental studies, we first undertook an examination of published protein expression data from a range of human and animal model studies including four of our own published datasets (Table 1; [6], [10]–[16]). With the increased stringency requirements for mass screening reporting, raw data sets are publicly available for many experimental comparisons, allowing further examination of the data for proteins of interest which may not have been highlighted in the full manuscript. We began with a focus on the expression levels of commonly used loading control proteins within the nervous system, as this is our specific area of research interest. We searched these datasets for expression data on a range of cytoskeletal proteins (actin, actinin and various tubulin isoforms), mitochondrial proteins (VDAC1 and VDAC2) and a nuclear protein (HC1), all of which are commonly used as a loading control or internal reference proteins for expression value normalisation in comparative protein quantitation experiments (Table 1).

**Table 1:** Examination of publicly available mass screening data reveals alterations in expression levels of commonly used “loading control” proteins.

Protein Name	Symbol	Protein ID	Localisation	Alteration	Model	Tissue	Technique	Reference
Actin	Actc1	IPI00654242.1	Cytoskeleton	Upregulated (3.07 fold)	SMA mouse	Cardiac Muscle	Label Free Proteomics	Mutsaers et al 2011 [6]
Actin	Acta1	IPI00110850.1	Cytoskeleton	Upregulated (3.01 fold)	SMA mouse	Skeletal Muscle	Label Free Proteomics	Mutsaers et al 2011 [6]
Actin (Unspecified Isoform)	-	p60710	Cytoskeleton	Absent	Wlds mouse	Striatal Synapses	2D Gel Proteomics	Wishart et al 2007 [10]
actinin, alpha 1	Actn1	Q7TPR4	Cytoskeleton	Downregulated (0.6 ratio)	SOD1 mouse	Cortical Synapses	iTRAQ Proteomics	Flynn et al 2012 [11]
Histone Cluster 1	HC1	NP_056601	Nuclear	Upregulated (1.3 ratio)	AOPE mouse	Sciatic Nerve	iTRAQ Proteomics	Comely et al 2011 [12]
Tubulin Alpha 1B Chain	ActA1B	IPI00117348.4	Cytoskeleton	Upregulated (1.32 fold)	SMA mouse	Skeletal Muscle	Label Free Proteomics	Mutsaers et al 2011 [6]
Tubulin Beta 4 Chain	TubB4	IPI00109073.5	Cytoskeleton	Downregulated (−1.16 ratio)	SMA mouse	Hippocampus	iTRAQ Proteomics	Wishart et al 2010 [13]
Tubulin beta-4 chain	Tubb4	N/A	Cytoskeleton	Downregulated (−0.28 ratio)	Ercc1 mouse	Hippocampal synapses	iTRAQ Proteomics	Vegh et al 2012 [14]
tubulin, alpha 4a	TubA4A	P68366	Cytoskeleton	Downregulated (−1.13 fold)	FTLD patient	Cerebellum	Gel Separation & Label free	de-Souza et al 2012 [15]
tubulin, beta 2a	Tubb2A	Q9CWF2	Cytoskeleton	Upnregulated (0.26 ratio)	SOD1 mouse	Cortical Synapses	iTRAQ Proteomics	Flynn et al 2012 [11]
tubulin, beta 2a	Tubb2A	Q9CWF2	Cytoskeleton	Upregulated (0.26 ratio)	FTLD patient	Prefrontal Cortex	Gel Separation & Label free	de-Souza et al 2012 [15]
Voltage Dependant anion Channel 1	VDAC1	q60932	Mitochondrial	Downregulated (−4.08 fold)	Wlds mouse	Striatal Synapses	2D Gel Proteomics	Wishart et al 2007 [10]
Voltage Dependant anion Channel 1	VDAC1	q60932	Mitochondrial	Upregulated (96.4%)	PPT1 mouse	Cortex	Quantitative Western Blot	Kielar et al 2009 [16]
Voltage Dependant anion Channel 1	VDAC1	q60932	Mitochondrial	Downregulated (−0.43 ratio)	SOD1 mouse	Cortical Synapses	iTRAQ Proteomics	Flynn et al 2012 [11]

Note: Alterations listed in table are in units published in the original manuscript or accompanying supplementary material.

This initial analysis revealed evidence for differential expression of all loading controls assessed using mass screening tools, including both -array technologies and proteomics. Discrepancies in expression of loading control proteins were detected across a diverse range of conditions, including Alzheimer’s disease, lysosomal storage disorders and the motor neurone disease Spinal Muscular Atrophy (SMA). Notably, this re-interrogation of published data also identified differential expression of loading control proteins across an assortment of tissues sampled, an issue of potential critical importance for subsequent data normalisation and post-omics validation.

### Actin and Tubulin are differentially Expressed in Pathologically-affected Tissue from a Mouse Model of SMA

**Figure 1:**
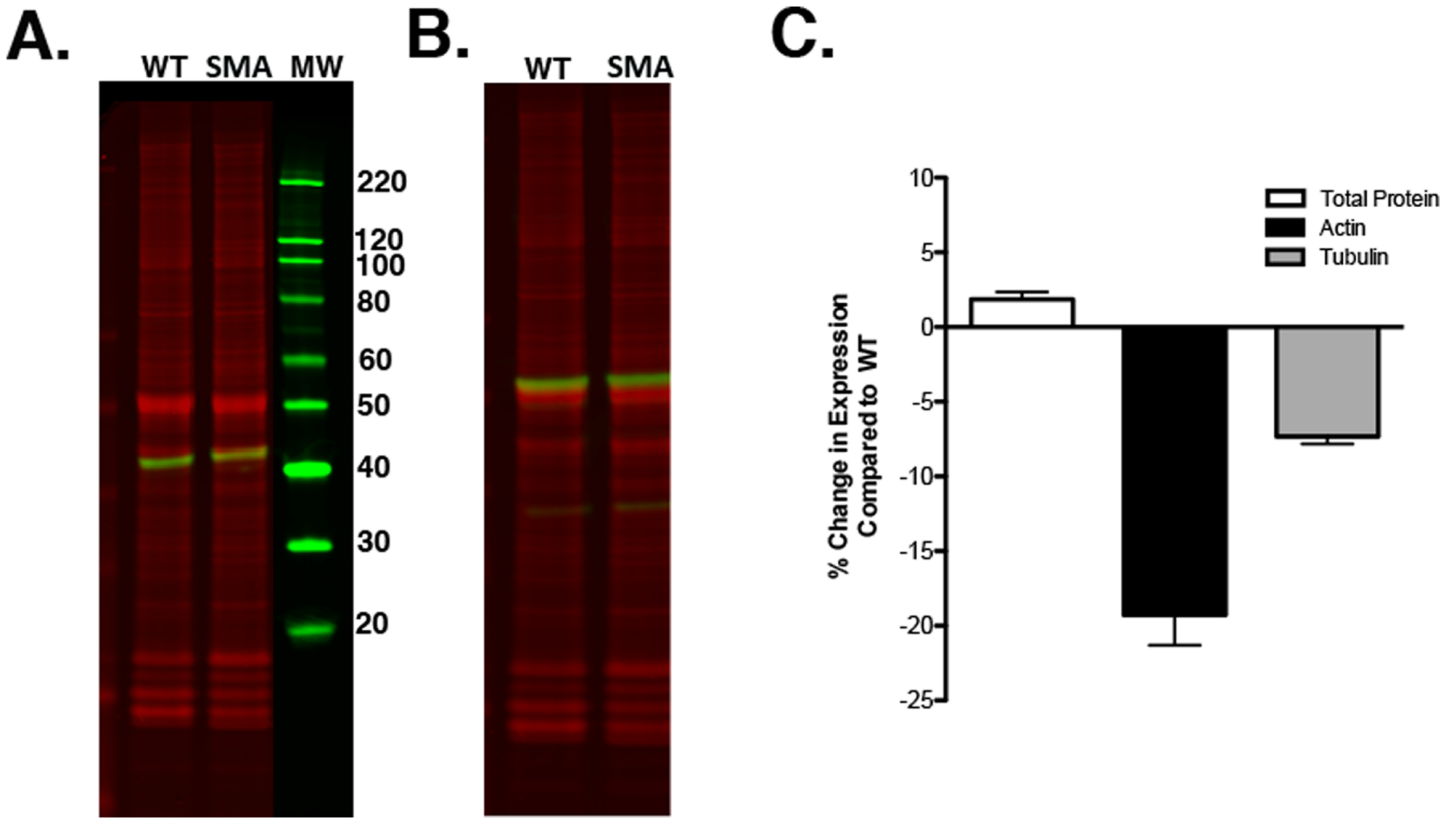
QWB for β-actin and β-tubulin demonstrates altered expression in pathological tissue. QWB for the commonly used loading controls actin and tubulin in SMA spinal cord extracts. A. Overlay of β-Actin QWB in green (42 kDa) with total protein stained gel (red). B. Overlay of β-tubulin QWB in green (60 kDa) with total protein stained gel (red). C. Quantification of percentage change in the expression levels of β-actin (black bar) and β-tubulin (grey bar) when comparing SMA mice (SMA) to wild-type controls (WT). Total protein stain (white bar) is used as a control.

Given that our analysis of raw data from published protein expression studies revealed widespread differential expression of common loading controls in various pathological conditions and neurological diseases ([10], [17], cf. Table 1), we next wanted to demonstrate that such alterations can also be detected when using modern QWB techniques. Using spinal cord tissue harvested from an established mouse model of severe SMA [18]–[19], we next quantified levels of β-actin and β-tubulin proteins. We found altered expression levels of both β-actin and β-tubulin when comparing the spinal cord of SMA affected mice (SMN:SMN2) with littermate controls (figure 1a. and 1b. respectively) using QWB. Both β-actin and β-tubulin expression were significantly down regulated in SMA compared to control tissue, by 19.3±2% and 7.3±0.5% respectively (mean ± SEM). Moreover, determination of total protein load demonstrated a high level of uniformity with a difference of 1.8±0.4% (mean ± SEM) between wild type and affected mice (n = 6) (figure 1c). Thus, altered LC protein expression as highlighted by proteomic studies on tissues from neurodegenerative conditions (including SMA; see Table 1) can also be detected by quantitative Western blot.

### Differential β-actin Expression is Detectable by QWB across Multiple Tissues from Wild-type Mice

**Figure 2:**
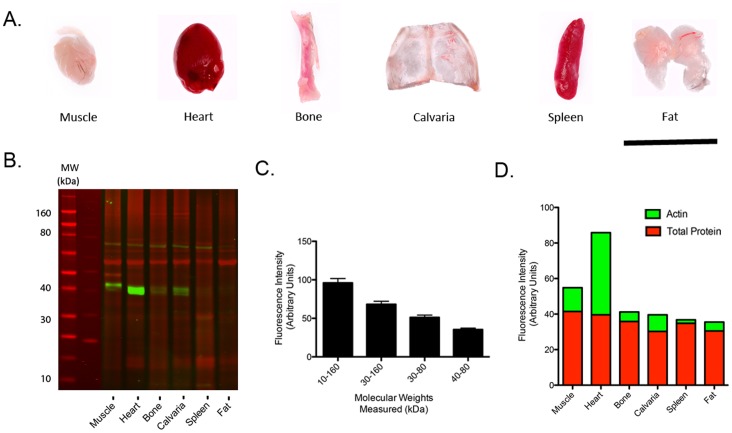
Comparative analysis of β-actin expression is highly variable across a broad range of tissues. A. Representative images of the tissue samples in which actin expression was assessed. From left to right: Muscle (Gastrocnemius), heart, bone (femur), calvaria, spleen and fat (gonadal). Scale bar = 1 cm. B. LICOR image of QWB demonstrating considerable variability of β-actin expression (green) in muscle, heart, bone, calvaria, spleen and fat extracts. Total protein stain gel image (red) is overlaid on QWB as a control. C. Total protein measurements for different molecular weight ranges demonstrates the accuracy of protein loading across the different tissue samples. D. Stacked bar graph demonstrating the comparative variability of β-actin (green bars) and total protein measurements (red bars) for each tissue examined.

Given that significant alterations in common loading control proteins can be detected in affected tissues sampled from a range of disease models (Table 1), we next wanted to establish whether similar differences in expression could be identified in healthy (‘wild-type’) tissue, and also establish whether several tissue types could be differentially affected within the same individual. This latter issue is particularly pertinent for many neurodegenerative diseases (including SMA), where multi-system pathology is now being reported (e.g. [20]). QWB screening of multiple tissues for expression of candidate proteins is common practice and is an especially critical procedure when comparing systemic protein expression profiles, identifying biomarkers for disease progression in peripherally accessible tissues, or validating regulatory proteins during genetic or pharmacological manipulation. It is therefore essential for a loading control to exhibit stable expression across a wide array of tissues. However, our studies using C57Bl/6 (‘wild-type’) mice demonstrated that expression of β-actin varied considerably across different tissues from the same mouse (figure 2 A & B). In order to verify that the variability of β-actin expression was not due to loading error, quantification of the protein load of each tissue was carried out on a series of mass ranges corresponding to protein electrophoretic migration (figure 2C). These data demonstrated low individual variation of protein load across the different tissue samples within the series of molecular weight ranges measured. Variability ranged from only 1.87% (SEM) in the 40–80 kDa range up to 5.65% for the broadest mass range of 10–160 kDa across all tissues. Therefore, consistency of load across each of the tissues was demonstrated and was independent of the mass range measured (figure 2C).

Expression of β-actin was strikingly different when comparing a diverse range of tissues (figure 2B & D). An appreciable disparity in β-actin expression was observed between heart and spleen tissue, with a difference of 44 arbitrary fluorescence units (AFU) contrasting with the consistent total protein load demonstrated in the 40–80 kDa molecular weight range (figure 2D). Critically, normalising data to β-actin expression for cross tissue comparisons could therefore result in skewing of data by up to 22 fold when comparing these tissues. However, there was some homology in β-actin expression between certain tissues such as bone and fat (5.33 AFU and 4.99 AFU respectively), and for these tissues it may therefore be acceptable to use β-actin as an internal control, but only when comparing these specific samples. Interestingly, a similar issue regarding β-actin has been raised with cross-tissue RNA expression profiling. For example, a qRT-PCR study in fish concurs with our findings suggesting that β-actin is not an ideal loading control for certain tissue comparisons particularly for heart, muscle and brain, whereas its expression is more consistent between kidney and spleen [9]. Our results therefore demonstrate that it would be advisable to use total protein expression as an indication of protein load to accurately perform a comparative expression analysis across a wide range of tissue samples.

### Loading Control Expression is not Homogeneous throughout Structurally Asymmetrical Tissues

**Figure 3:**
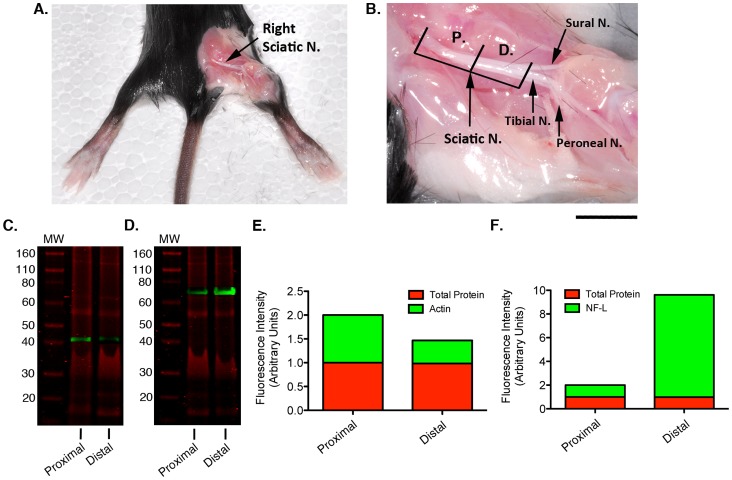
Actin & NF-L levels are not stable throughout different regions of the mouse sciatic nerve. Differential expression in proximal versus distal sciatic nerve preparations. A. Photograph depicting the lower half of a Bl6 mouse with sciatic nerve exposed on the right leg. B. Higher magnification photograph shows sciatic nerve and subsequent branches (anatomical nomenclature taken from [26]). Scale bar: A = 1 cm, B = 0.5 cm. C. Representative LICOR overlay image of β-actin QWB (green) and total protein stained gel (red) in proximal and distal portions of sciatic nerve from the same mouse. D. Representative LICOR overlay image of NF-L QWB (green) and total protein stained gel (red) in proximal and distal portions of sciatic nerve from the same mouse. E. Stacked bar graph demonstrating the comparative expression of β-actin (green bars) and total protein stain (red bars) expression in proximal and distal sections of sciatic nerve. F. Comparative expression of NF-L (green bars) and total protein stain (red bars) expression in proximal and distal sections of sciatic nerve.

Having demonstrated that expression of common loading controls such as actin was not necessarily consistent when comparing different tissues from the same animal, we next wanted to establish whether or not uniformity of protein expression was preserved throughout a single tissue from the same animal. Anecdotally it appears that researchers assume that loading control protein expression is stable within a given organ or tissue, with methodology sections of manuscripts routinely detailing gross, rather than specific, anatomical terms to describe tissue harvesting. To assess consistency within a biologically-relevant tissue, we measured and compared β-actin and neurofilament-light (NF-L) levels in proximal versus distal portions of the same mouse sciatic nerve using QWB. We found that levels of these LC proteins were not consistent throughout the two portions of the same nerve (Figure 3). Once again, we found that total protein load was consistent when quantified, however β-actin (a predominantly cytoplasmic isoform; [21] labelling was significantly higher (p = 0.0045), 52% greater ±14.3% (SEM), when comparing the proximal to distal portion of the sciatic nerve (figure 3C & E). In contrast expression of neurofilament (NF-L), a major component of the neuronal cytoskeleton [22], was appreciably higher, nearly 8 fold with up to a 4 fold SEM in the distal portion of the sciatic nerve (figure 3D & F). Therefore, these results stress that accuracy and consistency of dissection are crucial, even when evaluating the same tissues from a single animal for comparative analysis. Whilst this should be standard practice regardless, it is especially true for structurally asymmetrical tissues and failure to do so could have significant consequences for both -omics screens (such as proteomic comparisons) and for QWB analyses.

### Limitations in Standard Loading Controls: β-actin and β-tubulin Working Range and Sensitivity Explored

**Figure 4:**
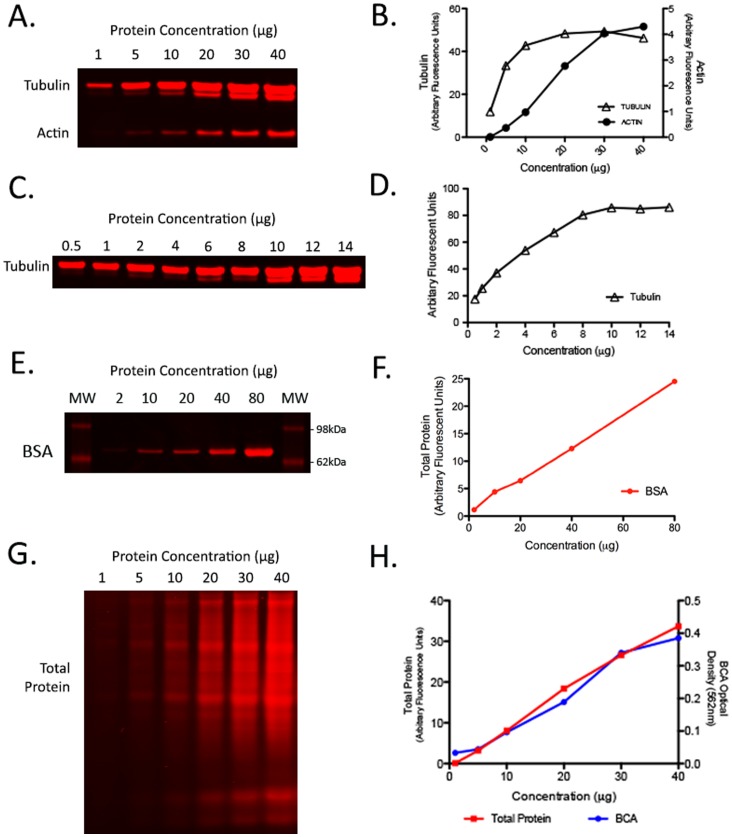
Linear range and sensitivity of total protein stain is greater than the conventional loading controls β-actin or β-tubulin. (A) Representative LICOR image for a protein dilution series of whole brain homogenate 1, 5, 10, 20, 30, 40 µg demonstrating the working range of β-actin and β-tubulin when using QWB. (B) Quantification of protein dilution series showing the linear ranges of β-actin (black circle) and β-tubulin (open triangle). Note that tubulin expression appears to saturate at less than 10 ug of brain homogenate. (C) In order to pinpoint the saturation level a tubulin specific protein dilution series over a smaller range (0.5, 1, 2, 4, 6, 8, 10,12 and 14 µg) establishing the saturation point of β-tubulin when using QWB. (D) Quantification of β-tubulin linear range. (E) Total protein stain of dilution series 2, 10, 20, 40, 80 µg made using the bovine serum albumin standard (2 µg/µl) from the Pierce BCA kit (see methods). BSA molecular weight is 66.5 kDa. Imaging of this dilution series demonstrates imaging of a broad concentration range without saturation at a single protein mass. (F) Graphical representation of quantification from BSA dilution series in panel E. This demonstrates wide linear detection and high correlation (0.998) validating the use of total protein measurements as a viable method for detecting protein load using the LICOR system. (G) Total protein stain of whole brain homogenate dilution series 1, 5, 10, 20, 30, 40 µg demonstrates the broad concentration range detectable without saturation. (H) Correlation between the total protein stain quantification (red line) and the BCA OD (blue line) for the protein dilution series demonstrates wide linear detection and high correlation (0.996 & 0.979 respectively) validating the use of total protein measurements as a viable “loading control” for QWB using the LICOR system.

The ideal internal loading control protein for QWB must be abundant with a wide linear range of detection to accommodate proteins of varying levels of expression. In order to evaluate the suitability of β-actin and β-tubulin as loading controls for QWB we tested both their working linear range and sensitivity by quantifying their expression throughout a dilution series, ranging from 1 to 40 µg of protein, produced from mouse whole brain tissue homogenate (figure 4a & b). The working range of β-actin where linearity was maintained was between 1 and 30 µg of protein loaded (figure 4a & b). Here, our quantitative Western blotting data conflicts with that of others who have reported that the linear range of β-actin was far smaller, only up to 2 µg of protein before saturation of the signal occurred [4], [8]. However these studies used less sensitive ECL detection methodologies therefore this disparity is most probably caused by the limitation of ECL based imaging and “quantification”.

When examining β-tubulin, it was evident that this protein was so abundant in brain extracts that the signal began to saturate out at less than 10 µg of protein (figure 4 a & b). In order to accurately determine the precise linear range of β-tubulin a tighter dilution series of protein load, 0.5 to 14 µg, was employed (figure 4c and d). Saturation of the β-tubulin signal occurred at 8–10 µg of protein load. Again, our QWB results conflicted with previous ECL studies suggesting that the linear range of β-tubulin peaks at 5 µg [4] and it is assumed that this is also likely due to the limitation of ECL based imaging and “quantification”. Our results suggest that β-tubulin rather than β-actin would be a more appropriate loading control when detecting low-abundance proteins in homogeneous extracts, however actin has a greater working range of sensitivity. In addition, our data has emphasised the superior sensitivity of fluorescent QWB in comparison to ECL as we demonstrate a far wider working range for both of these commonly used single protein loading controls.

### Total Protein Analysis is an Accurate Measure of Protein Load

Our analyses detailed above have highlighted several important variables that need considering when choosing an internal control for QWB, including but not limited to the linear range of sensitivity and disparities in expression across different tissue samples or within portions of the same tissue. Finally, we wanted to establish whether a total protein analysis approach would provide a more reliable and accurate measure of protein load for QWB experiments. To begin, we assessed the detection sensitivity using a dilution series created using a bovine serum albumin (BSA) protein standard (Figure 4E & F). BSA has a single band at a molecular weight of 66.5 kDa. We were able to detect a linear fluorescence profile across a broad concentration range as determined by the coefficient of variation (R^2^ value) of 0.998. Importantly, as the BSA standard dilution series effectively means that all of the protein loaded is represented by a single band, this not only validates the linear nature of detection using this system but also demonstrates the lack of saturation typically found with ECL based systems.

Finally, when applied to real biological samples using the LICOR Odyssey quantitative scanning system, total protein analysis (using coomassie) was linear in its detection across a broader range of protein loading than either actin or tubulin (1 to 40 ug; Figure 4G & H). The coefficient of variations for both Bicinchoninic Acid solution (BCA) assay and total protein analysis were 0.979 and 0.996 respectively. These R^2^ values demonstrated a high degree of linearity in both assays. Moreover, total protein analysis correlated directly with BCA protein concentration data (Figure 4G & H) throughout the broad 1–40 µg protein load dilution series further indicating its reliability as a control for protein load using the Odyssey quantitative imaging system. We therefore suggest that the use of total protein analysis provides a measure of protein load that circumvents many of the problems associated with the use of single loading control proteins: it is unchanged when comparing tissues from different models (c.f. Figure 1); it is consistent across different tissue types (c.f. Figure 2); and different portions of the same tissue (c.f. Figure 3).

## Conclusions

Western blotting has traditionally been a “semi-quantitative” technique using house keeping genes as internal reference standards. These standards are required to compensate for any technical errors that may have arisen due to issues such as poor transfer or unequivocal loading. However, our QWB studies have demonstrated a critical problem with the use of some common loading controls for this role. Differential expression of commonly used loading control proteins including β-actin and β-tubulin, occurs when comparing a wide range of tissues, when examining different portions of the same tissue and when pathological conditions arise. Therefore if normalisation (using single protein loading control expression as a correction factor) of quantitative Western blotting results is required, all of the resulting data could be skewed as a result of the differential expression of a single protein [23].

Total protein analysis is an alternative simple technique in QWB to accurately determine if equivalent protein loading has been achieved within a gel [24]. Data obtained by total protein analysis is independent of the pitfalls that can occur using “common” house keeping genes as loading controls. That is not to say that housekeeping genes cannot be used as loading controls, but that they should only be used in a limited fashion once the researcher has fully investigated sample expression homogeneity for the gene in question, and if the protein load falls within the working range of that particular loading control. Consequently, we propose it would be prudent to use total protein analysis to save time, resources, increase sensitivity and accuracy as well as the working range of protein load for quantitative Western blotting. Total protein analysis should therefore be considered an alternative standard reference for data normalisation in modern quantitative fluorescent Western blotting.

## Materials and Methods

### Tissue Harvesting and Protein Extraction

#### Ethics statement

All animal experiments were approved by a University of Edinburgh internal ethics committee and were performed under license by the UK Home Office (project license number 60/3891).

#### Preparation of severe model of Spinal Muscular Atrophy (SMA) spinal cord homogenates

Spinal cord were harvested from SMN/SMN2 severe model of SMA and wild-type controls at P5 and homogenised in RIPA buffer (Sigma, UK) containing 5% protease inhibitor cocktail (Roche) as previously described [13]. All SMA based comparisons were therefore Smn−/−;SMN2 (SMA) compared to wild-type (WT) litter mate controls unless otherwise stated. Protein was extracted and concentrations determined using a BCA assay (Pierce) according to manufacturers instructions, as previously described [10], [13], [25].

#### Preparation of range of tissue samples from C57/black mice

Quadraceps femoris muscle, gonadal fat, heart, calvaria, spleen and tibial bone tissue were dissected from 10 day old C57Bl/6 mice. Tissues were processed as outlined above.

#### Preparation of sciatic nerve tissue

Proximal and distal sections of the sciatic nerve from *C57/black mice* were dissected in 1xPBS (phosphate buffered saline) and frozen immediately on dry ice. The samples were homogenised in iTRAQ buffer (6 M urea, 2 M thiourea, 2% CHAPS, 0.5% SDS), sonicated 5×10 secs on ice, and centrifuged at 14 K for 30 minutes. 1∶100 dilutions of supernatant: dH_2_0 were used in a BCA assay following manufacturers instructions.

### Quantitative Western Blotting

Samples were denatured in NuPage® LDS Sample buffer 4X (Invitrogen, UK) at 98°C and 15 ug of protein loaded (with the exception of the protein dilution series) and run on commercially produced pre-cast 4–12% Bis-Tris gels (Invitrogen). Gels were run in duplicate in parallel in the same electrophoretic tank at the same time. One gel was stained using Instant blue (Expedeon) or coomassie (see total protein stain below) and one was used to transfer the protein to a polyvinylidene fluoride (PVDF) membrane using the I-Blot® transfer system (Invitrogen, UK) using programme 3 for 8.5 minutes. Membranes were incubated with Odyssey blocking buffer (Li-Cor) prior to incubation with rabbit polyclonal antibodies directed against β-actin (1∶1000, Abcam 8226), β-tubulin (1∶1000, Abcam 8226) and mouse monoclonal anti-NF-L (Millipore AB9568) overnight at 4°C. Goat anti-rabbit IgG (H+L) 800 CW, goat anti-rabbit (680 RD) and/or goat anti-mouse (H+L) was applied for 90 minutes at room temperature (1∶5000, LI-COR) prior to washing with PBS. Visualisation and quantification was carried out with the LI-COR Odyssey® scanner and software (LI-COR Biosciences). Blots (and gels) were imaged using an Odyssey Infrared Imaging System Scan resolution of the instrument ranges from 21 to 339 µm, and in this study blots (and gels) were imaged at 169 µm. Quantification was performed on single channels with the analysis software provided as previously described [10], [13], [25].

### Total Protein Gel Stain

All total protein stains within this manuscript have been carried out on gels and not membranes unless otherwise stated. As such there are caveats which should be taken into consideration if comparing total protein stained gels with membranes due to variables accompanying membrane transfer which are not accounted for when using this approach (see below). Post electrophoresis gels (see above) were stained using either Instant Blue (Expedeon) or Coomassie (0.1% Coomassie R250, 40% methanol, 10% acetic acid) solution. Gels were left in Instant blue for 1 hour and washed in dH_2_0 prior to visualisation. Coomassie stained gels were left in Coomassie solution for 1 hour and de-stained using several washes in de-stain solution (40% methanol, 10% acetic acid) and then washed in dH_2_0 prior to visualisation. Stained gels were imaged directly on the Li-COR Odyssey® scanner using the 700 channel and quantified using the Odyssey® software. See above. Incidentally the instant blue stain can be visualised in both the 700 and 800 channels however it has greater resolution in the 700 channel. For total protein stains to be of use as loading controls, ideally the membranes to be probed should be stained directly for load. However, there are limitations with this in modern fluorescent imaging systems. Coomassie is not as effective on most PVDF membrane types when compared to stained gels as the background auto-fluorescence is naturally higher. If used directly on a membrane coomassie does not strip to allow for re-probing in the same imaging channel. Ponceau stains are reversible but staining is difficult to visualise and remains to be proven linear in its adherence. Moreover, any stain which is not blue in presentation or does not fluoresce in the 680 or 800 wavelength channels used by the LICOR Odyssey scanner can not be imaged using this system and measurement scaling will therefore differ from the quantitative fluorescent blots for candidates of interest. Parallel commercially produced precast gels should therefore be used to reduce polyacrylamide matrix variability; they should be loaded at the same time using the same “master mix” (i.e. protein/water/loading dye) in the same tank (multi gel capacity) in order to be run from the same powerpack under the same conditions. A further variable when comparing a probed membrane to a stained gel are alterations introduced by variable transfer efficiency. Potential inconsistency in transfer efficiency generally occurs according to differential molecular weight rather than inter lane variability i.e. higher efficiency of transfer with lower molecular weight. The use of commercially procured transfer packs (such as the I-Blot® stacks; see above) coupled with a rapid semi-dry fast transfer system as detailed above should further improve reproducibility. By being aware of the steps where possible error could be introduced and taking the appropriate precautions such as those listed here, inter gel variability and transfer variance should be kept to a minimum, and appropriate data interpretation can be expected within the limitations of the system.

### Calculation of Linear Ranges of β-actin and β-tubulin

Concentration of protein extracts can be determined in a variety of ways. The most commonly used may be the Bicinchoninic Acid assays (BCA). BCA assays involve reduction of copper ions in a temperature dependant fashion with the level of reduction correlating with protein concentration. Reduced copper ions bind to BCA forming a purple product which can be detected at 562 nm. Each run includes a dilution series of a known protein standard – bovine serum albumin (BSA) as a reference curve to allow determination of absolute protein concentrations. As each reaction set can be subtly influenced by incubation time and temperature, samples which will be grouped together for analysis should routinely be assayed together against the same standard curve. Here we employed a series of protein dilutions (1, 5, 10, 20, 30, 40 µg) and (0.5, 1, 2, 4, 6, 8, 10, 12, 14 µg) which were produced from mouse whole brain homogenate. Preparation of the dilution series was carried out after the concentration of protein had been determined using a micro BCA assay (Pierce). Briefly, two 4–12% Bis-tris gels were loaded; one stained for total protein (Coomassie or Instant Blue) and the other was transferred for QWB as above. Visualisation and quantification was carried out using the Li-COR Odyssey imager and software. See above.

### Photography

Photographs of sciatic nerve dissection and organs were taken using a Nikon D200 camera with 105 mm micro NIKKIOR F2.8 lens.

### Data Analysis and Figure Production

QWB data was analysed using Odyssey software as per manufactures guidelines and as previously described [10]. Data was graphed and statistical comparisons carried out using GraphPad Prizm as previously described [25]. Image overlays were produced using Adobe photoshop to overlay 700 & 800 channels obtained from the Odyssey imager (LICOR Biosciences).

## References

[1] Zellner M, Babeluk R, Diestinger M, Pirchegger P, Skeledzic S, et al (2008) Fluorescence-based Western blotting for quantitation of protein biomarkers in clinical samples. Electrophoresis 29: 3621–3627. 18803224 10.1002/elps.200700935

[2] Gingrich JC, Davis DR, Nguyen Q (2000) Multiplex detection and quantitation of proteins on western blots using fluorescent probes. Biotechniques 29: 636–42. 10997278 10.2144/00293pf02

[3] Aoki K, Yamada M, Kunida K, Yasuda S, Matsuda M (2011) Processive phosphorylation of ERK MAP kinase in mammalian cells. Proc Natl Acad Sci USA 108: 12675–12680. 21768338 10.1073/pnas.1104030108 PMC3150946

[4] Suzuki O, Koura M, Noguchi Y, Uchio-Yamada K, Matsuda J (2011) Use of sample mixtures for standard curve creation in quantitative Western blots. Exp Anim 60: 193–196. 21512276 10.1538/expanim.60.193

[5] Wishart TM, Pemberton HN, James SR, McCabe CJ, Gillingwater TH (2008) Modified cell cycle status in a mouse model of altered neuronal vulnerability (slow Wallerian degeneration (Wlds). Genome Biol 9: R101 doi:10.1186/gb-2008-9-6-r101 18570652 10.1186/gb-2008-9-6-r101 PMC2481432

[6] Mutsaers CA, Wishart TM, Lamont DJ, Riessland M, Schreml J, et al (2011) Reversible molecular pathology of skeletal muscle in spinal muscular atrophy. Hum Mol Genet 20: 4334–4344. 21840928 10.1093/hmg/ddr360

[7] Castaño Z, Kypta RM (2008) Housekeeping proteins: Limitations as references during neuronal differentiation. The open access Neuroscience Journal 2: 36–40.

[8] Dittmer A, Dittmer J (2006) β-Actin is not a suitable loading control in Western blot analysis. Electrophoresis 27: 2844–2845. 16688701 10.1002/elps.200500785

[9] Dang W, Sun L (2011) Determination of internal controls for quantitative real time RT-PCR analysis of the effect of Edwardsiella tarda infection on gene expression in turbot (Scophthalmus maximus). Fish & Shellfish Immunol 30: 720–728. 21220029 10.1016/j.fsi.2010.12.028

[10] Wishart TM, Paterson JM, Short DM, Meredith S, Robertson KA, et al (2007) Differential proteomics analysis of synaptic proteins identifies potential cellular targets and protein mediators of synaptic neuroprotection conferred by the slow Wallerian degeneration (Wlds) gene. Mol Cell Proteomics 6: 1318–1330. 17470424 10.1074/mcp.M600457-MCP200 PMC2225590

[11] Flynn JM, Czerwieniec GA, Choi SW, Day U, Gibson BW, et al (2012) Proteogenomics of synaptosomal mitochondrila oxidative stress. Free Radic Biol Med 53: 1048–1060. 22796328 10.1016/j.freeradbiomed.2012.07.004 PMC3436120

[12] Comley LH, Fuller HR, Wishart TM, Mutsaers CA, Thomson D, et al (2011) ApoE isoform-specific regulation of regeneration in the peripheral nervous system. Hum Mol genet 20: 2406–2421. 21478199 10.1093/hmg/ddr147 PMC3098734

[13] Wishart TM, Huang JP, Murray LM, Lamont DJ, Mutsaers CA, et al (2010) SMN deficiency disrupts brain development in a mouse model of sever spinal muscular atrophy. Hum Mol Genet 19: 4216–4228. 20705736 10.1093/hmg/ddq340 PMC2951867

[14] Végh MJ, de Waard MC, van der Pluijm I, Ridwan Y, Sassen MJ, et al (2012) Synaptic proteome changes in a DNA repair deficient ercc1 mouse model of accelerated aging. J Proteome Res 1: 1855–1867. 10.1021/pr201203m 22289077

[15] Martins-de-Souza D, Guest PC, Mann DM, Roeber S, Rahmoune H, et al (2012) Proteomic analysis identifies dysfunction in cellular transport, energy, and protein metabolism in different brain regions of atypical frontotemporal lobar degeneration. J Proteome Res 11: 2533–2543. 22360420 10.1021/pr2012279

[16] Kielar C, Wishart TM, Palmer A, Dihanich S, Wong AM, et al (2009) Molecular correlates of axonal and synaptic pathology in mouse models of Batten disease. Hum Mol Genet 18: 4066–4080. 19640925 10.1093/hmg/ddp355 PMC2758138

[17] Gebhardt FM, Scott HA, Dodd PR (2010) Housekeepers for accurate transcript expression analysis in Alzheimer’s disease autopsy brain tissue. Alzheimer’s & Dementia 6: 465–474. 10.1016/j.jalz.2009.11.002 21044776

[18] Monani UR, Sendtner M, Coovert DD, Parsons DW, Andreassi C, et al (2000) The human centromeric survival motor neuron gene (SMN2) rescues embryonic lethality in Smn(−/−) mice and results in a mouse with spinal muscular atrophy. Hum Mol Genet 9: 333–339. 10655541 10.1093/hmg/9.3.333

[19] Murray LM, Comley LH, Thomson D, Parkinson N, Talbot K, et al (2008) Selective vulnerability of motor neurons and dissociation of pre- and post-synaptic pathology at the neuromuscular junction in mouse models of spinal muscular atrophy. Hum Mol Genet 17: 949–962. 18065780 10.1093/hmg/ddm367

[20] Hamilton G, Gillingwater TH (2013) Spinal muscular atrophy: going beyond the motor neuron. Trends Mol Med 19: 40–50. 23228902 10.1016/j.molmed.2012.11.002

[21] Dugina V, Zwaenepoel I, Gabbiani G, Clement S, Chaponnier C (2009) Beta and gamma-cytoplasmic actins display distinct distribution and functional diversity. J Cell Sci 122: 2980–2988. 19638415 10.1242/jcs.041970

[22] Hirokawa N, Glicksman MA, Willard MB (1984) Organization of mammalian neurofilament polypeptides within the neuronal cytoskeleton. J Cell Biol 98: 1523–1536. 6425303 10.1083/jcb.98.4.1523 PMC2113240

[23] Aldridge GM, Podrebarac DM, Greenough WT. Weiler IJ (2008) The use of total protein stains as loading controls: an alternative to high-abundance single protein controls in semi-quantitative immunoblotting. J Neurosci Methods 172: 250–254. 18571732 10.1016/j.jneumeth.2008.05.00 PMC2567873

[24] Welinder C, Ekbald L (2011) Coomassie as loading control in Western blot analysis. J Proteome Res 10: 1416–1419. 21186791 10.1021/pr1011476

[25] Wishart TM, Rooney TM, Lamont DJ, Wright AK, Morton AJ, et al (2012) Combining comparative proteomics and molecular genetics uncovers regulators of synaptic and axonal stability and degeneration in vivo. PLoS Genet 8: e1002936 doi:10.1371/journal.pgen.1002936 22952455 10.1371/journal.pgen.1002936 PMC3431337

[26] Silva DN, Coelho J, Frazílio Fde O, Odashiro AN, Carvalho Pde T, et al (2010) End-to-side nerve repair using fibrin glue in rats. Acta Cir Bras 25: 158–162. 20305882 10.1590/s0102-86502010000200007

